# Mutational and large deletion study of genes implicated in hereditary forms of primary hyperparathyroidism and correlation with clinical features

**DOI:** 10.1371/journal.pone.0186485

**Published:** 2017-10-16

**Authors:** Elena Pardi, Simona Borsari, Federica Saponaro, Fausto Bogazzi, Claudio Urbani, Stefano Mariotti, Francesca Pigliaru, Chiara Satta, Fabiana Pani, Gabriele Materazzi, Paolo Miccoli, Lorena Grantaliano, Claudio Marcocci, Filomena Cetani

**Affiliations:** 1 Department of Clinical and Experimental Medicine, University of Pisa, Pisa, Italy; 2 Endocrinology Unit, Department of Medical Sciences and Public Health, University of Cagliari, Cagliari, Italy; 3 Department of Surgical, Medical and Molecular Pathology and Critical Area, University of Pisa, Pisa, Italy; 4 Department of Medical Sciences, Hospital Villa Albani, Anzio (RM), Italy; 5 University Hospital of Pisa, Endocrine Unit 2, Pisa, Italy; Odense University Hospital, DENMARK

## Abstract

The aim of this study was to carry out genetic screening of the *MEN1*, *CDKN1B* and *AIP* genes, both by direct sequencing of the coding region and multiplex ligation-dependent probe amplification (MLPA) assay in the largest monocentric series of Italian patients with Multiple Endocrine Neoplasia type 1 syndrome (MEN1) and Familial Isolated Hyperparathyroidism (FIHP). The study also aimed to describe and compare the clinical features of *MEN1* mutation-negative and mutation-positive patients during long-term follow-up and to correlate the specific types and locations of *MEN1* gene mutations with onset and aggressiveness of the main MEN1 manifestations. A total of 69 index cases followed at the Endocrinology Unit in Pisa over a period of 19 years, including 54 MEN1 and 15 FIHP kindreds were enrolled. Seven index cases with MEN1 but *MEN1* mutation-negative, followed at the University Hospital of Cagliari, were also investigated. FIHP were also tested for *CDC73* and *CaSR* gene alterations. *MEN1* germline mutations were identified in 90% of the index cases of familial MEN1 (F-MEN1) and in 23% of sporadic cases (S-MEN1). *MEN1* and *CDC73* mutations accounted for 13% and 7% of the FIHP cohort, respectively. A *CDKN1B* mutation was identified in one F-MEN1. Two *AIP* variants of unknown significance were detected in two *MEN1*-negative S-MEN1. A *MEN1* positive test best predicted the onset of all three major MEN1-related manifestations or parathyroid and gastro-entero-pancreatic tumors during follow-up. A comparison between the clinical characteristics of F and S-MEN1 showed a higher prevalence of a single parathyroid disease and pituitary tumors in sporadic compared to familial MEN1 patients. No significant correlation was found between the type and location of *MEN1* mutations and the clinical phenotype. Since all *MEN1* mutation-positive sporadic patients had a phenotype resembling that of familial MEN1 (multiglandular parathyroid hyperplasia, a prevalence of gastro-entero-pancreatic tumors and/or the classic triad) we might hypothesize that a subset of the sporadic MEN1 mutation-negative patients could represent an incidental coexistence of sporadic primary hyperparathyroidism and pituitary tumors or a MEN1 phenocopy, in our cohort, as in most cases described in the literature.

## Introduction

Familial primary hyperparathyroidism (PHPT) may be part of complex syndromes, i.e. multiple endocrine neoplasia (MEN) type 1, 2A, and 4, or occur as an isolated (non-syndromic) disorder (FIHP), inherited as autosomal dominant traits. MEN1 is characterized by the combined occurrence of multiple endocrine tumors, namely parathyroid glands hyperplasia with an almost complete penetrance by the age of 50, gastro-entero-pancreatic (GEP) neuroendocrine tumors (NETs), and anterior pituitary tumors. A minority of patients develop a wide spectrum (more than 20) of endocrine and non-endocrine associated manifestations other than the classic endocrinopathies (i.e. adrenal cortical tumors, foregut carcinoid tumors, angiofibromas, collagenomas, and cutaneous or visceral lipomas), accounting for the variable phenotypic presentations.

Sporadic MEN1 defines patients fulfilling the diagnostic criteria of MEN1, but without a family history of MEN1-related manifestations. Patients with one of the major MEN1-related manifestations associated with less common MEN1 tumors are defined as “phenocopy variants” or atypical MEN1.

Inherited loss-of-function mutations in the tumor suppressor *MEN1* gene (11q13), the most common molecular defect causing MEN1, have been detected in about 70–80% and 30% of patients with familial and sporadic MEN1, respectively [1]. *MEN1* mutations are scattered throughout the entire coding sequence of the gene, with no mutational hot spots. Since the cloning of the *MEN1* gene, more than 1,500 germline and somatic mutations have been reported [2,3]. Large monoallelic deletions encompassing multiple exons or the whole gene are responsible for a subset of MEN1 patients with no *MEN1* gene mutations using the Sanger sequencing analysis (10–20%) [4–8]. According to the *two-hit* hypothesis, a somatic loss of heterozygosity at 11q13 accounts for the acquisition of a homozygous recessive state at tissue level in a dominantly inherited cancer susceptibility syndrome.

The clinical variability between patients carrying the same *MEN1* mutation, as well as between members of the same kindred, raises the hypothesis that modifier genes and/or epigenetic tumour-predisposing events might also have a role in the pathogenesis of MEN1 [9].

A subset of FIHP kindreds also carried germline *MEN1* mutations. In addition, loss-of-function mutations of the *Cell Division Cycle 73* (*CDC73*) or of the *Calcium-Sensing receptor* (*CaSR*) genes and, very recently, gain-of-function mutations of the *Glial Cells Missing Homolog 2* (*GCM2*) gene, have also been detected in a few FIHP kindreds [10].

MEN4 is a syndrome characterized by the same clinical heterogeneity and tumor spectra of MEN1, but also in a few cases by gonadal, adrenal, renal, and thyroid tumors. MEN4 is caused by germline mutations of the *Cyclin Dependent Kinase Inhibitor 1B* (*CDKN1B*) gene, codifying for p27^kip1^, an inhibitor of cyclin-dependent kinases, involved in the negative control of cell cycle progression [11]. A kindred carrying a *CDKN1B* germline mutation, formerly classified as FIHP, has subsequently been considered a MEN4 case [12].

Germline mutations of the *Aryl-hydrocarbon Interacting Protein* (*AIP*) gene, the gene responsible for some sporadic pituitary adenomas, and a subset (20%) of Familial Isolated Pituitary Adenoma (FIPA), have been detected in a few cases of sporadic PHPT and one MEN1 kindred [13–15].

The aim of this study was to fully describe the clinical manifestations of the largest series of Italian patients with sporadic and familial MEN1 syndrome and FIHP mostly followed in a single-center and to screen them for *MEN1* gene abnormalities in order to find the best predictor of a *MEN1* positive test. In addition, patients with negative *MEN1* gene testing were screened for mutations of the *CDKN1B*, *AIP* genes (MEN1 patients) or *CDKN1B*, *AIP*, *CDC73* and *CaSR* genes (FIHP patients). We also compared the clinical characteristics of familial and sporadic MEN1 and sought for a correlation between *MEN1* mutations and the clinical phenotype.

## Materials and methods

### Patients

The study was reviewed and approved by the University Hospital of Pisa Ethics Committee, and in accordance with the Declaration of Helsinki. All participants provided signed informed consent to participate in the study.

A total of 69 index cases with hereditary form of PHPT followed at the Endocrine Unit of Pisa from 1997 to 2015, including 54 MEN1 and 15 FIHP kindreds, were enrolled in the study (Pisa cohort) [16–18]. The MEN1 cohort also included 62 affected relatives. In addition, seven index cases of MEN1 syndrome with no *MEN1* gene mutations, followed at the University Hospital of Cagliari Endocrine Unit, were also included in the study (Cagliari cohort).

According to the international guidelines, MEN1 patients were classified as follows: i) familial MEN1 (F-MEN1) by the presence in the proband of at least two MEN1 major lesions, with a first-degree relative with at least one major lesion; ii) sporadic MEN1 (S-MEN1) in the absence of family history for MEN1-related manifestations; iii) atypical MEN1 by the association of a single major lesion with one or more uncommon MEN1-related manifestations [19].

The diagnosis of GEP-NETs, bronchial and thymic NETs was made by histological and immunohistochemical examinations of resected specimens according to the World Health Organization (WHO) criteria [20,21]. Of note, the 2015 WHO classification categorized all NETs of the lung and thymus as malignant independently of whether metastases were present or not [21]. The diagnosis of parathyroid and pituitary carcinomas was made according to the 2004 WHO criteria [22].

The provisional diagnosis of FIHP was based on: i) evidence of PHPT in the proband and in at least one first degree relative; ii) finding of an abnormal parathyroid gland at histology: iii) absence of MEN1 manifestations other than PHPT at baseline and during long-term follow-up, after an extensive clinical, instrumental and biochemical evaluation.

### Gene nucleotide sequence analyses (*MEN1*, *CDKN1B*, *AIP*, *CDC73* and *CaSR* genes)

DNA was extracted from index patients’ peripheral leucocyte with Maxwell16 Instrument according to the manufacturer’s instructions (Promega Corp., Madison, USA). The entire coding region and intron/exon boundaries of the *MEN1* gene (GenBank entry NM_130799.2) were firstly investigated by sequencing germline DNA from all patients. DNA was PCR-amplified and sequencing reactions on both strands were performed with BigDye Sequencing Reaction kit v.1.1 (Applied Biosystems, Foster City, CA) and separated on ABI 3130XL automatic sequencer (Applied Biosystems). When we did not identify any *MEN1* mutations or large deletions (see below), we carried out the sequence analyses of the DNA coding regions of *CDKN1B/*p27^Kip1^ (NM_004064.4) and *AIP* (NM_ 003977.3) genes. In FIHP probands, in addition to the screening of the *MEN1*, *CDKN1B* and *AIP* genes, we directly sequenced the entire coding region and intron/exon boundaries of the *CDC73* (NM_024529.4) and *CaSR* (NM_000388.3) genes. In kindreds carrying *MEN1* mutation, the mutational analysis of the region of interest was extended to first degree relatives of the proband, independently of the presence of MEN1-related signs and symptoms. By analyzing the DNA of both parents of a mutation-positive sporadic case we could assess the *de novo* origin of the mutation.

### Multiplex ligation-dependent probe amplification (MLPA) assay

To detect large monoallelic deletions or amplifications in *MEN1*, *AIP* and *CDKN1B* genes not detected by conventional sequencing techniques, we performed MLPA analysis. Two different MLPA kits, the SALSA MLPA probemix kit P244-B1 and P244-C1 (MRCHolland, Amsterdam, The Netherlands), were used and experiments were performed according to the manufacturer’s instructions, as previously reported [23]. Three reference DNA blood samples from healthy subjects and a negative control (sample without DNA), as well as appropriate positive controls, were included in all experiments.

### Statistical analysis

All analyses were carried out in the Pisa cohort of patients. The association between F-MEN1 and S-MEN1 or *MEN1* mutation-positive and negative patients and some dichotomous variables (gender, single vs multiglandular parathyroid disease, presence or absence of MEN1-related tumors) was determined using Chi-square or Fisher’s exact tests, according to the sample size (Chi-square calculation was used when all expected cell frequencies were ≥ 5). Chi-square or Fisher’s exact tests were also used to test the association between tumor type and aggressiveness in the group of *MEN1* mutation-positive patients. Mean age at first clinical manifestation of different categories of patients was compared using the *t* test. A value of P<0.05 was considered statistically significant.

### Systematic review of the literature

#### Publication search

We have conducted a review of the literature of reported cases with sporadic MEN1 syndrome. We performed a systematic literature search in PubMed from inception to January 2017 using the key word “MEN1 syndrome AND mutation”. We did not use more specific terms such as “non-familial”, “sporadic” or “isolated” to restrict the search, because in some cases they did not appear in the title, in the list of key words, or in the abstract of the articles. No limitations were placed on the language of publication or type of study. All eligible studies were retrieved and their bibliographies were checked for other relevant publications.

#### Inclusion criteria

Studies eligible were published as a full text and included: i) assessment of the mutational state of the *MEN1* gene; ii) description of the phenotype of all individuals studied. These studies included either case series or single case reports. When the same author or group reported results from the same patient population in more than one article, the most informative was included.

#### Data extraction

Information was carefully extracted from all eligible studies. The following data were collected from each study: bibliographic reference, number of patients analyzed, number of patients with or without *MEN1* mutations, MEN1-related phenotype.

## Results

### Clinical characteristics

#### MEN1 cohort

The Pisa cohort included 54 probands, 34 females (63%) and 20 males (37%) (female to male ratio of 1.7:1), with a mean age at first manifestation of 45 (SD 14, range 19–71 yr). Thirty-one (57%) patients were classified as F-MEN1 and 22 (41%) as S-MEN1. We did not classify the remaining patient as familial or sporadic because she was adopted. The prevalence of various MEN1-associated tumors and non endocrine manifestations in the probands, with the exclusion of the adopted patient, is summarized in Table 1. The classical triad of MEN1-related tumors was present in 17 of 54 (31%) probands, PHPT and pituitary tumors in 11 (20%) and PHPT and GEP tumors in 23 (43%). Two patients with S-MEN1 had an atypical presentation.

**Table 1:** Tissue-selectivity of tumors in familial and sporadic MEN1 probands.

*Hormone-secreting tumours*	*Familial MEN1*		*Sporadic MEN1*		*P value*^*a*^
	*Patients (n = 31)*		*Patients (n = 22)*		
**PHPT**	31/31	(100%)	21/22	(95%)	0.42
As first manifestation	27/31	(87%)	14/22	(64%)	
Parathyroidectomy	28/31	(90%)	17/21	(81%)	
*Uniglandular*	1/28	(4%)	8/17	(47%)	
					**0.0008**
*Multiglandular*	27/28	(96%)	9/17	(53%)	
**Gastro-entero-pancreatic tumours**	27/31	(87%)	12/22	(54%)	**0.008**
As first manifestation	4/31	(13%)	1/22	(5%)	0.39
*Non-Functioning*	21^b^/27	(78%)	10/12	(83%)	
					0.70
*Functioning*	7^b^/27	(26%)	2/12	(17%)	
• Gastrinoma	4/7	(57%)	0/2	(0%)	0.44
• Insulinoma	2/7	(29%)	1/2	(50%)	1
• Glucagonoma	1/7	(14%)	1/2	(50%)	0.42
**Anterior pituitary tumours**	14/31	(45%)	14/22	(64%)	0.18
As first manifestation	0.31	(0%)	3/22	(14%)	0.07
*Non-Functioning*	6^c^/14	(46%)	9^d^/14	(64%)	
					0.27
*Functioning*	9^c^/14	(64%)	6^d^/14	(46%)	
• Prolactinoma	6^c^/9	(67%)	1/6	(17%)	0.27
• Somatotrophinoma	2/9	(22%)	3/6	(50%)	0.58
• Gonadotrophinoma	0/9	(0%)	1^d^/6	(17%)	0.43
• Somatomammotrophinoma	1/9	(11%)	1/6	(17%)	1
**Adrenal tumors**	13/31	(42%)	7/22	(32%)	0.78
*Non-Functioning*	13/13	(100%)	4/7	(57%)	
					**0.03**
*Cortisol-secreting adenoma*	0./13	(0%)	3/7	(43%)	
**Carcinoids**	3/31	(10%)	5/22	(23%)	0.25
• Thymic	1/3	(33%)	0/5	(0%)	
					0.38
• Bronchial/pulmonary	2/3	(67%)	5/5	(100%)	
***Other non-endocrine manifestations***					
Vascular tissue neoplasms (angioma, angiofibromas)	5/31	(16%)	3/22	(14%)	1
Gastric leiomyoma	1/31	(3%)	0/22	(0%)	1
Uterine fibromatosis in female	5/19	(26%)	2/14	(14%)	0.67
Lipoma	10/31	(32%)	3/22	(14%)	0.20
Meningioma	1/31	(3%)	0/22	(0%)	1
Neuroblastoma	1/31	(3%)	0/22	(0%)	1
Breast cancer in female	1/19	(5%)	2/14	(14%)	0.60
Thyroid cancer	2/31	(6%)	0/22	(0%)	0.50

^***a***^Statistical significance was determined by Fisher or Chi square test; level of significance, P<0.05. P values<0.05 have been reported in bold characters.

^b^One patient has both a non-secreting GEP-NET and an insulinoma

^c^One patient has both a PRL-secreting adenoma and a NF microadenoma

^d^One patient has both a FSH-secreting adenoma and a NF microadenoma

The phenotype of the entire cohort (probands and relatives, n = 116, female to male ratio of 1.5:1) was characterized by PHPT and pituitary and GEP tumors in 27 patients (23%), PHPT and GEP tumors in 51 (44%), PHPT and pituitary tumors in 14 (12%). PHPT and pituitary tumors with or without minor tumors were present in 22 (19%) and 2 patients (2%), respectively. There was no gender difference in the occurrence neither of each tumor type nor in the combination of MEN1-associated tumors in all affected patients. In the whole group, 285 tumors were diagnosed (2.3 tumors per patient). PHPT was present in 114/116 (98%) patients; 81 (71%) of them underwent surgical neck exploration. Eighty-eight percent of patients had the excision of more than one parathyroid gland either at first or with repeated surgery. Seventeen of the 116 patients (15%) had malignant tumors: 7 pancreatic NETs (5 non-functioning and 2 gastrinomas), 10 carcinoids (1 thymic and 9 bronchials), 1 parathyroid and 1 pituitary carcinoma. Seven of 17 (41%) patients had distant metastases of gastrinomas and non-functional GEP tumors, thymic and bronchial carcinoids [lymph nodes (n = 5), lung (n = 1) and liver (n = 3)]. In the remaining 10 patients the diagnosis of malignancy was only based on histological criteria [20–22].

The Cagliari cohort included 7 MEN1 probands (5 females and 2 males) with a mean age of 50 years (SD 12). One patient had F-MEN1 (multiglandular PHPT and non-functioning pancreatic NET) and three S-MEN1 (two with uniglandular PHPT associated with a prolactin-secreting adenoma, and one with multiglandular PHPT associated with a glucagonoma). Three additional sporadic cases had an atypical MEN1 [pituitary adenomas (one TSH-secreting and 2 non-functioning) associated with adrenal tumors (one cortisol-secreting and 2 non-functioning)].

#### Familial MEN1 (n = 31)

**Fig 1:**
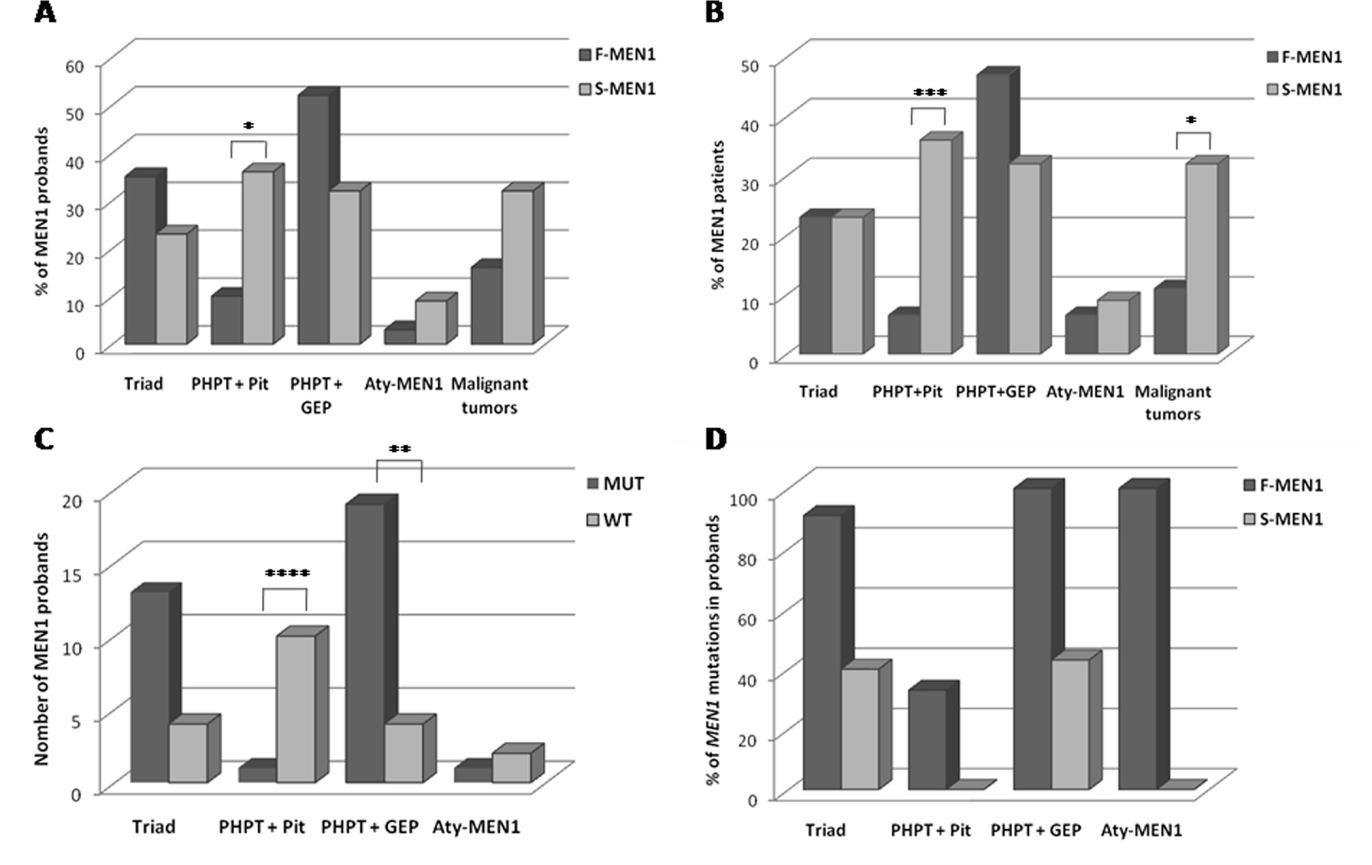
MEN1 manifestations in the groups of F-MEN1, S-MEN1 probands and *MEN1* mutation-negative and mutation-positive probands. (**A**) Association of the main MEN1-related tumors and tumor aggressiveness in probands with F-MEN1 and S-MEN1 syndrome. (**B)** Association of the main MEN1-related tumors and tumor aggressiveness in patients of the whole cohort with F-MEN1 and S-MEN1 syndrome. (**C)** Association of the main MEN1-related tumors in *MEN1* mutation-positive and mutation-negative probands. **(D)** Detection rate of *MEN1* gene mutations within each main clinical presentation in *MEN1* mutation-positive probands with and without family history. Aty-MEN1 refers to atypical MEN1. Statistical significance was determined by Fisher or Chi-square test. *P<0.05, **P<0.01, ***P<0.001, ****P<0.0001.

The index cases group included 19 females (61.3%) and 12 males (38.7%) (female to male ratio of 1.6:1), with a mean age at first clinical manifestation of 43 (SD 13, range 19–68 yr). The phenotype of the probands is shown in Fig 1A.

This cohort also included 62 affected relatives (36 females and 26 males, F:M 1.4:1). In 6 relatives the age at first clinical manifestation was not available. The mean age was 37 (SD 16, range 13–67 yr). Ten (16%) patients had PHPT associated with pituitary and GEP tumors, 3 (5%) PHPT and pituitary tumors, 28 (45%) PHPT and GEP tumors, 20 (32%) PHPT with or without minor tumors, one (2%) pituitary adenoma and bronchial carcinoid.

PHPT represented the first endocrine manifestation of the disease in 27 (87%) of the index cases. Non-secreting pituitary and GEP tumors were the most common neoplasia, whereas prolactinomas and gastrinomas were the most frequent functioning pituitary and GEP tumors, respectively (Table 1). There was no gender difference in the presentation of the MEN1-associated tumors.

#### Sporadic MEN1 (n = 22)

This cohort included 11 females (50%) and 11 males (50%) (female to male ratio of 1:1), with a mean age at first clinical manifestation of 46 (SD 14, range 23–71 yr). PHPT associated with pituitary and GEP tumors was present in 5 (23%) patients, PHPT and pituitary tumors in 8 (36%), and PHPT and GEP tumors in 7 (32%) (Fig 1A). Two patients (9%) had an atypical presentation, namely a bronchial carcinoid associated in one case with a GH-secreting pituitary adenoma and in the other with a single parathyroid adenoma. PHPT represented the first endocrine manifestation of the disease in 64% of the index cases. Non-secreting pituitary and GEP tumors were the most common neoplasia, whereas GHoma, insulinoma or glucagonoma the most frequent functioning pituitary and GEP tumors (Table 1). No gender difference in the presentation of the MEN1-associated tumors was observed.

#### Comparison between the phenotype of F-MEN1 and S-MEN1

The female to male ratio and the mean age at first clinical manifestation did not differ between the two cohorts (P = 0.41 and P = 0.44, respectively).

A single parathyroid adenoma was more frequent in S-MEN1 compared to F-MEN1 probands, whereas the opposite occurred when considering parathyroid hyperplasia (P<0.001, Table 1). The statistical significance increased to P<0.0001 when the analysis included relatives submitted to parathyroidectomy (PTx) (n = 35). GEP tumors were more frequently observed in the group of F-MEN1 compared to S-MEN1 probands (P<0.01). The occurrence of non-functioning adrenal lesions was similar in both groups, whereas cortisol-secreting adenomas were only present in S-MEN1 (P<0.05, Table 1).

The various combinations of tumors at diagnosis did not differ between F-MEN1 and S-MEN1 probands, except for the co-occurrence of PHPT and pituitary tumors that were more frequent in S-MEN1 (P = 0.04) (Fig 1A). When the analysis was performed in the whole cohort (probands and relatives), the association of PHPT and pituitary tumors, as well as the presence of malignant tumors, was significantly more common in patients with S-MEN1 than with F-MEN1 (P<0.001 and P = 0.02, respectively) (Fig 1B). The two groups did not differ for other clinical parameters.

#### FIHP kindreds (n = 15)

This cohort included 15 probands, 14 females (93%) and 1 male (7%) (female to male ratio of 14:1), with a mean age of 47 (SD 13, range 14–67 yr). This ratio decreased to 2.7:1 when the affected relatives (n = 22, 13 females and 9 males) were included. Twenty-five patients (14 probands and 11 relatives) underwent PTx. Thirteen (52%) had a single parathyroid adenoma and 12 (48%) a multiglandular parathyroid hyperplasia.

### Genetic analyses

#### Patients with MEN1 syndrome

Thirty germline *MEN1* mutations were identified by direct sequencing, 25 (81%) in familial cases. The majority of the mutations were located in exons 2 (33%), 9 (22%) and 10 (22%). No mutations were detected in exons 4, 5, 6 and 8 (Table 2). The most common recurrent mutations (common to 2 or 3 index cases) were Met1?, c.249_252delGTCT, c.628_631delACAG, c.784-9G>A, Arg415Stop and c.1676delA (Table 2). Twenty-two out of 30 (73%) were frameshift, nonsense or splice site junction mutations, leading to a truncated protein (Table 2). Of note, two probands carried the c.1A>G substitution, affecting the ATG start codon and creating an unclassified variant (Met1?). All but one (Lys201Stop) mutations have already been reported. The Lys201Stop nonsense mutation at exon 3 was identified in a 36-year-old woman affected by persistent PHPT after subtotal PTx, pituitary PRL-secreting microadenoma treated with cabergoline and a bronchial carcinoid cured by left superior lobectomy. The patient did not develop other endocrine tumors during an 8-year follow-up. Her 10-year-old unaffected daughter also harbor the mutation.

**Table 2:** *MEN1* gene mutations detected in 54 probands with MEN1 syndrome.

Nucleotide annotation	Mutation type	Protein annotation	Number of carriers	Familial (F)/ Sporadic (S)
**Exon 2**				
c.1A>G	?	p.Met1?	2	F
c.87delA	fs	p.Arg29ArgfsX80	1	F
c.207_208insGCCCC	fs	p.Pro69ProfsX52	1	F
c.249_252delGTCT	fs	Leu83LeufsX35	3	2 F 1 S
c.286C>T	ns	p.Gln96Stop	1	F
c.134A>G	ms	p.Glu45Gly	1	F
c.415C>A	ms	p.His139Asn	1	F
**Exon 3**				
c.601A>T	ns	p.Lys201Stop	1	F
c.628_631delACAG	fs	p.Thr210SerfsX13	2	1F 1S
**Intron 4**				
c.784-9G>A	sp		3	F
**Exon 7**				
c.957C>A	ns	p.Tyr319Stop	1	F
c.1031C>G	ms	p.Thr344Arg	1	F
**Exon 9**				
c.1213C>T	ns	p.Gln405Stop	1	S
c.1243C>T	ns	p.Arg415Stop	2	F
c.1252G>A	ms	p.Asp418Asn	1	F
c.1331T>C	ms	p.Leu444Pro	1	F
c.1354C>T	ms	p.Arg452Trp	1	F
**Exon 10**				
c.1382_1389dup8	fs	p.Ala464ArgfsX98	1	F
c.1546_1547insC	fs	p.Arg516ProfsX15	1	F
c.1579C>T	ns	p.Arg527Stop	1	F
c.1666G>T	ns	p.Glu556Stop	1	F
c.1676delA	fs	p.Lys559ArgfsX3	2	1F 1S
**Large deletion**				
-	ld	5'UTR-exons 1–2	1	S
-	ld	exons 9–10	1	F
-	ld	whole gene	2	F

fs = frameshift; ms = missense; ns = nonsense; sp = splice-site; ld = large deletion

MLPA analysis also identified four *MEN1* large deletions: a deletion spanning the whole gene in 2 F-MEN1, a deletion of exons 9 and 10 in one F-MEN1, and a deletion encompassing the 5’UTR region, exons 1 and 2 in one S-MEN1.

In summary, mutations of *MEN1* gene were identified in 28 of 31 (90%) F-MEN1 and in 5 of 22 (23%) S-MEN1 (P<0.0001). The index case who was adopted carried a nonsense mutation in exon 7 (Tyr319Stop). The mutations identified in all index cases were also identified in the affected relatives and in 6 unaffected young relatives (healthy carriers). DNA from the parents was only available in one mutated S-MEN1 case. Both tested negative, so we could confirm the existence of a *de novo* mutation.

A frameshift mutation of the *CDKN1B* gene [c.374_375delCT (Ser125Stop)] was detected in one MEN1 proband of the Cagliari cohort [15].

Two germline missense variants in exon 1 of the *AIP* gene [Arg9Gln (c.26G>A) and Arg16His (c.47G>A)] were found in two S-MEN1 probands. Both variants have already been described [24–31].

The remaining MEN1 families and sporadic cases, including the Cagliari cohort cases, were negative at the screening of *MEN1*, *CDKN1B* and *AIP* genes.

#### Patients with FIHP

Two different *MEN1* mutations (Asp418His, c.824+1G>A) and one *CDC73* germline mutation (c.131+1G>A) in three unrelated FIHP kindreds were identified and already reported [16–18].

No mutations of the *MEN1*, *CDC73*, *CaSR*, *AIP* or *CDKN1B* genes nor large deletions in *MEN1*, *AIP* and *CDKN1B* genes were found in the remaining FIHP cases.

### Comparison between phenotype and genotype

In this analysis we compared the phenotypes of *MEN1*-positive and *MEN1*-negative probands (n = 54) and the whole cohort (n = 122), independently of whether they were classified as familial or sporadic (Table 3).

**Table 3:** Clinical characteristics of the patients (probands and whole cohort) according to *MEN1* genotype.

			Probands (n = 54)			Total cohort (n = 122)		
			*MEN1*+	*MEN1-*	P value^a^	*MEN1*+	*MEN1-*	P value^a^
**Patients**			34	20		98	24	
**Mean age at diagnosis (SD)**			42 yr (12)	49 yr (15)	**0.02**	37 yr (15)*	49 yr (15)*	**<0.001**
**Female**			20	14	0.41	56	17	0.22
**Male**			14	6		42	7	
**F:M**			1.4:1	2.3:1		1.3:1	2.4:1	
**Tumors (N)**			102	52		229	56	
**Tumor rate per patient**			3.0	2.6		2.3	2.3	
**PHPT**			34 (100%)	19 (95%)	0.37	91 (93%)	23 (96%)	1
*PTx*			32	14		66	15	
• Multiglandular			32	5	**<0.0001**	65	6	<**0.0001**
• Uniglandular			0	9		1	9	
**GEP tumors**			32 (94%)	8 (40%)	**<0.0001**	70 (71%)	8 (33%)	**<0.001**
*Non-functioning*			25^b^	7	0.66	57^c^	7	0.7
*Functioning*			8^b^	1		15^c^	1	
• Gastrinoma			4	0	1	10	0	0.37
• Insulinoma			2	1	0.33	3	1	0.25
• Glucagonoma			2	0	1	2	0	1
**Pituitary tumors**			14 (41%)	15 (75%)	**0.02**	28 (30%)	19 (79%)	**<0.0001**
*Non-functioning*			9^d^	7^e^	0.48	20^d^	8^e^	**0.04**
*Functioning*			6^d^	9^e^		9^d^	12^e^	
• Prolactinoma			5	2	**0.04**	8	3	**<0.01**
• Somatotrophinoma			0	5	0.09	0	6	**0.02**
• Gonadotrophinoma			0	1	1	0	1	1
• Somatomammotrophinoma			1	1	1	1	2	1
**Carcinoid tumors**			5 (15%)	3 (15%)	1	7 (7%)	3 (13%)	0.41
• Thymic			1	0	1	1	0	1
• Bronchial/Pulmonary			4	3	1	6	3	1
**Adrenal tumors**			15 (41%)	5 (25%)	0.16	30 (31%)	6 (25%)	0.59
*Non-functioning*			14	4	0.44	29	4	0.07
*Functioning*			1	1		1	2	
**Malignant tumors**					0.73	7 (7%)	5 (21%)	0.06

*MEN1*+ = *MEN1* mutation-positive; *MEN1*- = *MEN1* mutation-negative

^a^Statistical significance was determined by Fisher or Chi square test; level of significance, P<0.05. P values <0.05 have been reported in bold characters.

^b^One patient has both an insulinoma and a non-functioning GEP tumor.

^c^Two patients has both an insulinoma and a non-functioning GEP tumor.

^d^One patient has both a prolactinoma and a non-functioning pituitary tumor.

^e^One patient has both a gonadotrophinoma and a non-functioning pituitary tumor.

*The age at first clinical manifestation of 6 relatives was not available.

*MEN1*-mutated probands and patients of the whole cohort were significantly younger than *MEN1*-negative ones (P<0.05 and P<0.001, respectively). The female to male ratio did not differ between the *MEN1*-positive and *MEN1*-negative probands and whole cohort (Table 3).

The rate of association between PHPT and pituitary tumors was significantly lower (P<0.0001) in *MEN1*-positive than in *MEN1*-negative probands. Conversely, the rate of association between PHPT and GEP tumors was significantly higher (P<0.01) in *MEN1*-positive than in *MEN1*-negative probands (Fig 1C). Fourty-six probands and 35 relatives underwent PTx with the excision of a single or multiple pathological parathyroid glands. Multiglandular parathyroid disease was present in all *MEN1* mutation-positive probands and in 33/34 (97%) relatives. Conversely, a single parathyroid adenoma, whose excision resulted in the cure of PHPT, was found in 9/14 (64%) and 9/15 (60%) *MEN1* mutation-negative probands and patients of the whole cohort submitted to PTx, respectively [mean follow-up 7yr (SD 4)] (P<0.0001 in both groups) (Table 3).

A total of 154 and 285 MEN1-related tumors occurred in the group of probands and in the whole cohort, respectively. A GEP tumor occurred in 74% of probands and in 64% of patients of the cohort. In both groups, they were predominantly non-functioning (78% and 80%, respectively). Fifty-four percent of probands and 39% of all patients developed almost a pituitary tumor (62% non-functioning and 38% functioning, respectively). The distribution of functioning GEP tumors between *MEN1*-negative and positive cases in both groups was not statistically different. The rate of GH-secreting adenomas was significantly lower (P<0.05) in *MEN1*-positive than in *MEN1*-negative patients of the cohort, whereas the rate of prolactin-secreting adenomas was significantly higher in *MEN1*-mutated than MEN1-negative probands and cohort (P<0.05 and P<0.01, respectively) (Table 3). The rates of tumors per patient (2.3) and malignant MEN1-related tumors did not differ between *MEN1*-positive and *MEN1*-negative patients of the whole cohort.

In the whole *MEN1*-positive cohort, the rate of mutations in exon 2 was significantly higher (P = 0.03) in patients with pituitary adenomas than in those with other MEN1-related tumors, whereas the rate of mutations in exon 9 was significantly lower (P = 0.02) in patients without pituitary tumors (Table 4). There was no difference in the clinical phenotype between patients carrying *MEN1* large deletions or point mutations (Table 4). There was no significant association (P = 0.34) between malignant tumors and the type of mutation, i.e. truncated (frameshift, nonsense, large deletions and splice site mutations) vs non-truncated mutations (missense mutations), excluding the Met1? mutation, whose protein effect is still unknown, in the group of probands or in the whole cohort (Table 4). When the detection rate of *MEN1* gene mutations in the 53 probands was correlated with the clinical presentation of the syndrome and family history, the presence of mutations in the patients with triad was found in 91% and 40% of familial and sporadic cases, respectively. Thirty-three percent of familial probands with the co-occurrence of PHPT and pituitary tumors, but any proband with S-MEN1 with the same phenotype carried *MEN1* mutations. All F-MEN1 probands with PHPT and GEP tumors were mutated, while 43% of S-MEN1 probands with PHPT and GEP tumors carried a mutation (Fig 1D).

**Table 4:** Correlation analysis between the sites and the type of MEN1 mutations and the occurrence of different tumors in *MEN1*-mutation positive probands and relatives.

		n	PHPT	Pituitary tumors	GEP tumors	Carcinoids	Adrenal tumors	Malignant tumors
Probands		34	34 (100%)	14 (41%)	32 (94%)	5 (15%)	15 (44%)	7 (21%)
Cohort		98	91 (93%)	28 (29%)	70 (71%)	7 (7%)	30 (31%)	12^ (12%)
***Site of mutation***								
**Exon 2**	Probands	10	10 (100%)	5 (50%)	10 (100%)	1 (10%)	4 (40%)	1 (10%)
	Cohort	21	20 (95%)	**10 (48%)**^**a**^	19 (91%)	3 (14%)	6 (29%)	3^ (14%)
**Exon 3**	Probands	3	3 (100%)	2 (67%)	2 (67%)	2 (67%)	2 (67%)	2 (67%)
	Cohort	7	5 (71%)	3 (43%)	3 (43%)	2 (29%)	3 (43%)	2 (29%)
**Intron 4**	Probands	3	3 (100%)	1 (33%)	3 (100%)	0 (0%)	0 (0%)	0 (0%)
	Cohort	9	9 (100%)	2 (22%)	7 (78%)	0 (0%)	1 (1%)	0 (0%)
**Exon 7**	Probands	2	2 (100%)	2 (100%)	2 (100%)	0 (0%)	1 (50%)	0 (0%)
	Cohort	4	4 (100%)	2 (50%)	3 (75%)	0 (0%)	2 (50%)	0 (0%)
**Exon 9**	Probands	6	6 (100%)	2 (33%)	6 (100%)	0 (0%)	2 (33%)	1 (17%)
	Cohort	26	23 (88%)	**3 (12%)**^**a**^	18 (69%)	0 (0%)	6 (23%)	3 (11%)
**Exon 10**	Probands	6	6 (100%)	1 (17%)	5 (83%)	1 (17%)	3 (50%)	2 (33%)
	Cohort	17	17 (100%)	5 (29%)	10 (59%)	1 (6%)	6 (35%)	2 (12%)
**Large deletion**	Probands	4	4 (100%)	1 (25%)	4 (100%)	1 (25%)	3 (75%)	1 (25%)
	Cohort	14	13 (93%)	3 (21%)	10 (71%)	1 (7%)	6 (43%)	2 (14%)
Mutation type*								
**Truncating**^**§**^	Probands	26*	26 (100%)	12 (46%)	24 (92%)	4 (15%)	14 (54%)	6 (23%)
	Cohort	66*	62 (94%)	23 (35%)	47 (71%)	6 (9%)	24 (36%)	10^#^ (15%)
**Non-truncating**°	Probands	6*	6 (100%)	2 (33%)	6 (100%)	1 (17%)	1 (17%)	1 (17%)
	Cohort	28*	25 (89%)	3 (11%)	20 (71%)	1 (4%)	6 (21%)	2^#^ (7%)

^a^ P<0.05

^§^Frameshift, nonsense, whole/partial gene deletions and splice site alterations

°Missense mutations

^^^ 2 patients have a carcinoid and a metastatic gastrinoma

^#^ 1 patient has a carcinoid and a metastatic gastrinoma

*M1? variant has neither been counted in the truncating nor in the non-truncating mutations

### Systematic review

#### Results of the search

The search retrieved a total of 892 references. We screened all records and applied exclusion criteria based on the title/abstract or full text revision. Eight hundred thirty-three studies did not meet the inclusion criteria, because: i) the study population consisted in F-MEN1; ii) *MEN1* gene was not analyzed. From the 59 potentially relevant articles, we further excluded 14 studies due to: i) unavailability of the exact number of patients genetically tested; ii) lack of clinical features of the patients. Forty-five studies were eligible and included in the systematic analysis in S1 Table.

## Discussion

The aim of our study was to extend the knowledge on the phenotype of hereditary PHPT and highlight differences between the clinical characteristics of sporadic and familial MEN1 and *MEN1*-positive and *MEN1*-negative patients.

We report the clinical and genetic data of the largest cohort of consecutive MEN1 patients, both F-MEN1 and S-MEN1, followed at a single Italian institution collected over a period of 19 years. Previous studies of four single-center experiences included 32, 7, 20 and 12 MEN1 index cases [32–35]. Two of these studies [32,35] enrolled consecutive patients, whereas the others included patients selected for the presence of thymic and GEP tumors [33,34]. A very recent study, aimed at developing an Italian nationwide multicenter registry/database of MEN1 syndrome, describes the clinical, biochemical, and genetic data of 475 cases. The cases were collected in our Institution and 14 referral centers for endocrine inherited tumors and MEN syndromes [36].

In our study, PHPT, which is commonly the earliest manifestation of the disease, was present in 98% of all probands, GEP tumors in 70% and pituitary adenomas in 52%. These figures are in agreement with those of other studies [1]. At variance with the data reported in the literature, non-functioning adenoma was the most common pituitary tumor (<5% vs 55%) in our cohort, with no significant difference between the familial and sporadic MEN1 probands (Table 1). We might speculate that this unusually high percentage of non-functioning pituitary tumors could be due to a referral bias, since our Endocrine Unit is also a referral center for pituitary disease. A percentage similar to ours (42%) was found in a large Dutch MEN1 cohort (n = 323). In this study, a systematic quarterly screening during follow-up of pre-symptomatic pituitary tumors, using magnetic resonance imaging, gave rise to a significant increase in non-functioning tumors [37]. In our cohort prolactinoma was the most common secreting pituitary tumor (22% of all pituitary tumors and 50% of all functioning lesions) and non-functioning pancreatic NETs were the most common (80%) GEP neoplasms, gastrinomas being the main functioning lesion (40%). Insulinomas accounted for one third of all functioning GEP tumors and was frequently associated (75%) with other non-functioning GEP-NETs.

The rate of a single parathyroid tumor and cortisol-secreting adrenal tumors significantly differed between S-MEN1 and F-MEN1 probands. Tissue-selectivity of pituitary tumors was evident in the group of S-MEN1compared to the F-MEN1 patients (both probands and relatives) (64% vs 29%, P = 0.002).

### Correlation between phenotype and *MEN1* genotype in the overall cohort

Familial and sporadic cases of MEN1 are genetically indistinguishable since germline mutations are present in both cases. The so-called ‘sporadic’ MEN1 case is usually caused by a *de novo* mutation that can be transmitted to the progeny and will be considered the index case of a novel MEN1 family. In our cohort, a *de novo* origin of the mutation was assessed only in one case due to DNA unavailability of the parents from the remaining cases. The occurrence of a *de novo MEN1* mutation in S-MEN1 reported so far has been estimated at 10%, but parental DNA was only studied in about 3% of cases. A true incidence rate of *de novo* mutations cannot therefore be established [2].

In our cohort, we identified a molecular alteration of the *MEN1* in 90% of F-MEN1. *MEN1* mutations, scattered throughout the entire coding region of the gene, were present in 81% of cases. Mutational hot spots were not identified. Large germline deletions were present in a further 10% of cases. Truncated mutations, including large deletions, were the most common mutations, according to the literature (76% vs 74%) [2]. Three of the 5 most recurrent mutations (common in 2 or 3 index cases) were located in three of the 9 sites (I-IX), identified by Lemos et al., where germline mutations mostly occur (c.249_252delGTCT in site I, c.628_631delACAG in site IV and c.784-9G>A in site V) [2].

To date, no definitive correlation between *MEN1* germline mutation and the phenotype has been convincingly established [2]. A correlation between mutations encoding a truncated menin and aggressive tumors, such as thymic, bronchial carcinoids, or metastatic GEP tumors, has been found by some but not all authors [33,38–40]. In our cohort there was no significant association between truncating vs missense mutations, nor between large deletions vs point mutations and malignant tumors [41]. Interestingly, the two whole gene deletions found in our cohort were carried by two kindreds in which one of the relatives developed a malignant tumor (a thymic carcinoid with lymph node and lung metastases in one case and a non-functioning grade 3 pancreatic neuroendocrine carcinoma in the other), while the other relatives had a benign course of the disease (Table 5). So far, 17 *MEN1* large deletions (5 whole and 12 partial gene deletions) have been reported in 14 F-MEN1, 2 S-MEN1 and one FIHP (Table 5). Only 4 familial and 1 sporadic MEN1 cases showed an aggressive phenotype. Therefore, to date, no definitive conclusions on the correlation between large deletions and a more severe phenotype can be drawn. We found an association between the location of *MEN1* mutations and the occurrence of specific tumor types. In agreement with Kouvaraki et al., mutations in exon 2 were significantly more frequent in patients with pituitary adenomas than in those with other MEN1-related tumors. Mutations in exon 9, however, occurred mostly in patients without pituitary involvement [42].

**Table 5:** Literature studies investigating *MEN1* gene large deletions using different analysis methodologies.

Authors	Analyzed Patients/ Kindreds	Patients/Kindreds with deletion (affected members)	Deletion type	Case index phenotype	MEN1-associated tumors of carriers members	Analysis methodology	Age at diagnosis index case (younger affected relative)
*Kishi et al* [84]*Kikuchi et al*. [85]	1 F-MEN1	1 F-MEN1(3)	Whole gene (68 kbp)	Typical MEN1	na	Gene dosage assay	na
*Bergman et al*. [86]	12 F-MEN1	1 F-MEN1 (na)	Partial (5'UTR-exon 5)	PHPT, GEP(I), PIT (NF)	na	Southern blot	na
	8 S-MEN1						
	7 MEN1-LIKE						
	5 FIHP						
	1 FIPA						
*Cavaco et al*. [87]	6 F-MEN1	F-MEN1 1 (6)	Partial (exon 7–3'-UTR)	PHPT, GEP(G), **TC**	PHPT (4), GEP (3: 1 NF, 1 G, 1 **K**), 1 PIT (NF)	Southern blot	41 (32)
		F-MEN1 2 (3)	Partial (5'-UTR-exon 9)	PHPT, PIT (NF), **LC**	PHPT (2), PIT (2, NF), 1 GEP (GL)		56 (41)
*Cebrian et al*. [49]	27 F-MEN1	F-MEN1 (4)	na	PIT (1 PRL, 1 NF), GEP PHPT	na, PHPT	Southern blot	63 (na)
	27 S-MEN1						
	1 FIHP	FIHP (5)	na				35 (na)
*Lairmore et al*. [45]	9 F-MEN1	1 F-MEN1 (3)	Partial (5'UTR-exon 2)	na	na	Southern blot	na
*Fukuuchi et al*. [88]	1 F-MEN1	F-MEN1 (3)	Whole gene (29 kbp)	PHPT. GEP(G), PIT (NF)	PHPT (2), PIT (PRL)	Gene dosage assay	41 (na)
*Tham et al*. [89]	34 F-MEN1	F-MEN1 1 (na)	Partial (exons 1–10)	na	na	MLPA	na
	59 S-MEN1	F-MEN1 2 (na)	Partial (exons 8–10)	na	na		na
*Owens et al*. [5]	23 F-MEN1	1 S-MEN1 (1)	Partial (intron 4- exon 6; c.784-105_910del312)	PHPT, GEP(G), PIT (NF)	na	MLPA	54
	78 S-MEN1						
*Cosme et al*. [90]	1 F-MEN1	1 F-MEN1 (10)	Partial (exons 1–2)		PHPT (8), GEP(3: G), PIT (2, PRL)	MLPA	49 (18)
*Rusconi et al*. [47]	1 S-MEN1	1 S-MEN1 (1)	Whole gene (>22.3 kbp)	PHPT, GEP(NF), PIT (PRL)	na	aCGH	11
*Raef et al*. [46]	1 F-MEN1	1 F-MEN1 (7)	Whole gene (4,8 kbp)	PHPT, GEP(**Met I**)	GEP(5)	MLPA and aCGH	30 (17)
*Zatelli et al*. [91]	1 F-MEN1	1 F-MEN1 (4)	Partial (exons 1–3)	PHPT, GEP(GL), PIT (PRL)	PHPT (3), GEP(2: 1 **Met NF K**, 1 **Met I**), PIT (3: 2 PRL, 1 ACTH), 1 **BC**	TaqMan Copy Number Variation assay and MLPA	56 (na)
*Manoharan et al*. [92]	1 S-MEN1	1 S-MEN1 (1)	Whole gene (5,6 kbp)	PHPT, GEP(NF **K**), PIT (NF)	na	MLPA and aCGH	na
*Present Study*	31 F-MEN1	F-MEN1 1 (5)	Whole gene	PHPT, GEP(NF), **Met TC**	PHPT (3), GEP(3: NF, 1 **K**), PIT (2: NF)	MLPA	56 (13)
	22 S-MEN1	F-MEN1 2 (6)	Whole gene	PHPT, GEP(NF)	PHPT (5), GEP(3: NF) PHPT (1)		60 (14)
		F-MEN1 3 (2)	Partial (exons 9–10)	PHPT, GEP(NF), PIT(NF)	PHPT (1)		54 (na)
		1 S-MEN1 (1)	Partial (5'UTR-exon2)	PHPT, GEP(GL)			34

F-MEN1 = Familial MEN1, S-MEN1 = Sporadic MEN1, PHPT = Primary hyperparathyroidism, GEP = Gastro-entero-pancreatic tumor, PIT = Pituitary adenoma, G = Gastrinoma, I = Insulinoma, GL = Glucagonoma, NF = Non-functioning tumor, PRL = Prolactinoma, ACTH = Adreno-corticotrophic hormone-sectreting tumor, Met = Metastatic, K = Carcinoma, TC = Thymic carcinoid, LC = Lung carcinoid, BC = Bronchial carcinoid, MLPA = Multiplex-ligation-dependent probe amplification, na = Not available.

As expected, *MEN1* mutations strongly segregated within the F-MEN1 group (P<0.0001). They were identified only in 5 (23%) of S-MEN1, a percentage very similar to that found by some authors (25–29%) [43,44], but lower than the average percentage of all reported cases (n = 829, 42%) (S1 Table). Interestingly, one of the five mutations was a deletion in the *MEN1* encompassing the promoter and exons 1–2, previously reported in two MEN1 kindreds [45,46]. Large deletions were found in two other S-MEN1 cases [35,47]. All S-MEN1 mutation-positive patients had a clinical phenotype similar to that of F-MEN1, presenting multiglandular parathyroid hyperplasia, GEP tumors and, in one case, a non-functioning pituitary macroadenoma. For these reasons, we have focused our analysis mainly on the difference between *MEN1*-positive and negative groups, independently of their family history.

The index cases that scored as negative at *MEN1* mutation testing developed MEN1 manifestations later in life than *MEN1*-positive patients, as in line with the literature [48]. The co-occurrence of parathyroid and pituitary tumors represented the hallmark of mutation-negative genotype, whereas 97% of *MEN1*-positive probands presented parathyroid and GEP tumors or the association of the three main MEN1-related tumors. Of note, PHPT in *MEN1*-negative patients was mainly due to uniglandular parathyroid involvement. Interestingly, although there was no significant difference in the genotype between functioning and non-functioning pituitary tumors, all patients with GH-secreting adenoma were *MEN1-*mutation negative, whereas most patients carrying prolactinomas (78%) were mutation-positive [49].

### Correlation between phenotype and *MEN1* genotype in the sporadic and atypical cohorts

In order to better characterize the clinical phenotype of S-MEN1 we carried out an accurate revision of the case series and case reports described in the literature (S1 Table). We found 45 different studies that included 466 sporadic cases both *MEN1*-positive (33%) and *MEN1*-negative (67%). The most common phenotype was the co-occurrence of PHPT and pituitary tumors (41%); 21% had PHPT in combination with GEP-NETs, 19% had PHPT associated with pituitary tumors and GEP-NETs, 4% had GEP-NETs and pituitary tumors. Interestingly, 86% of all index cases with PHPT and pituitary tumors were *MEN1* mutation-negative, whereas 69% of patients with PHPT associated with pituitary tumors and GEP-NETs were *MEN1* mutation-positive (S1 Table). The finding in our cohort of S-MEN1 cases that all patients with PHPT and pituitary tumors were *MEN1*-negative, suggests that some cases, incorrectly diagnosed as S-MEN1, might have an incidental coexistence of sporadic PHPT and pituitary tumor, as both diseases frequently occur in the general population. The higher percentage of uniglandular parathyroid disease in S-MEN1 mutation-negative compared to S-MEN1 mutation-positive patients (77% vs 0%, P = 0.02) confirms previous observations.

Both S-MEN1 cohorts from Pisa and Cagliari included 5/28 (18%) cases classified as atypical. Sixty-seven cases of suspicious/atypical or MEN1-related cases have been reported in the literature in studies in which the *MEN1* genetic analysis was performed (S1 Table). Atypical MEN1 represents a group of cases which do not strictly fulfill the clinical criteria for MEN1 diagnosis but might be suspicious for MEN1 since they have the association of one major with one or more minor MEN1-related tumors. In such cases the current clinical practice guidelines recommend *MEN1* gene mutation screening [19]. Patients developing multiple parathyroid tumors before the age of 30, gastrinomas or multiple islet cell tumors, and FIHP are also classified as atypical MEN1 cases. In our study, the five cases of atypical S-MEN1 did not carry *MEN1* mutations in. This finding is in agreement with the observation that 82% of the atypical S-MEN1 cases reported in the literature were negative for *MEN1* mutations (S1 Table). Interestingly, all cases of PHPT or pituitary adenomas associated with adrenal tumors were *MEN1*-mutation negative whereas, respectively, 60% and 20% of PHPT or pituitary combined with carcinoids, were *MEN1*-mutation positive. This finding confirms that adrenal tumors associated with only one of the three major MEN1-related tumors and without family history of MEN1 have a low predictive value for a positive *MEN1* mutation test [50]. Bronchial and thymic carcinoids had a prevalence of 8.2% in all affected MEN1 patients of this study (10/122), both with typical and atypical phenotype, in line with that reported in the literature (3.6–8.4%) [51]. In contrast to sporadic adrenal tumors, where the prevalence of an underlying MEN1 syndrome is <1%, the prevalence of thymic carcinoids in the setting of a MEN1 syndrome is 25%. This suggests that the co-occurrence of carcinoids with one of the main MEN1-related tumor may represent a true MEN1.

### Non-*MEN1* genetic anomalies in the overall cohort

To determine whether other genetic alterations might be involved in the remaining MEN1-negative cases, we extended the investigation to other genes recently associated with MEN1-related disorders.

Menin, the protein product of *MEN1* gene, directly regulates the expression of a number of target genes, among which is the Cyclin-Dependent Kinase Inhibitor 1B (*CDKN1B*), codifying for p27^Kip1^ [52,53]. Germline mutations of the *CDKN1B* gene are responsible for MEN4 syndrome and their prevalence in 400 patients affected by MEN1-related states is 2.5% [54]. We identified a *CDKN1B* gene mutation c.374_375delCT (Ser125Stop) in one F-MEN1 of the Cagliari cohort affected by a multiglandular PHPT and multiple GEP tumors [15]. Four of the 15 different germline *CDKN1B* variations so far identified were detected in patients with F-MEN1, while the remaining were detected in patients with FIPA, FIHP, S-MEN1 or patients with sporadic PHPT or acromegaly [15,54–56].

Aryl-hydrocarbon interacting protein (*AIP*) gene is the major gene responsible for the predisposition to pituitary adenomas, mainly in the setting of Familial Isolated Pituitary Adenoma (FIPA) (20%). Germline *AIP* mutations have also been identified among young sporadic patients with pituitary macroadenomas or gigantism (8–20%) [14] in a S-MEN1 index case with acromegaly and recurrent PHPT, and in 2 of 132 apparently sporadic parathyroid adenomas [13,57]. Herein we detected two N-terminal *AIP* variants—Arg9Gln and Arg16His -already reported in two S-MEN1 *MEN1*-negative patients. The patient with Arg9Gln had multiglandular PHPT, non-functioning pancreatic NET and a mixed prolactin and GH-secreting pituitary adenoma. Interestingly, tumors co-secreting GH and prolactin were common in *AIP* mutation-positive patients [58]. Arg9Gln variant has been identified in 3 unrelated young sporadic patients affected by GH, prolactin and ACTH-secreting adenomas respectively, the latter being a condition rarely associated with *AIP* mutations [28,30]. Neither of the studies attributed a definite pathogenic role to the variant. Although Arg9Gln has been reported in the dbSNP database as a very low frequency polymorphism (rs139459091) and *in silico* analysis has predicted a benign change, recent *in vitro* analyses have demonstrated that the variant had a reduced protein stability and caused a de-regulation of the wild type protein on cAMP pathway, increasing GH secretion [59].

The *AIP* variant Arg16His that we identified in an S-MEN1 patient with uniglandular PHPT and insulinoma, has previously been reported in several FIPA families and patients with apparent sporadic pituitary adenomas, as well as in 2 colorectal cancers. However, its identification in a few healthy subjects, as well as in the normal tissue surrounding a tumor harboring the somatic mutation, and the lack of segregation with the disease in some families, suggests that this change might be considered a variant of unknown significance or a rare polymorphism [26–29,59–61].

### Genetic profiles in FIHP

A systematic literature review on FIHP from the first report in 1936 to date documents 238 FIHP kindreds with a family history of surgically-treated PHPT in first-degree relatives, with clinical and biochemical exclusion of MEN1 and HPT-JT [10, 62–83]. Genetic testing of *MEN1*, *CDC73* and *CaSR* has been carried out in 153 FIHP kindreds and mutations of these genes have been identified in 27%, 15% and 6.7%, respectively. Recently, a study performed on 40 FIHP index cases detected two gain-of function missense variants in the *GCM2* gene, coding for a transcription factor with a pivotal role in parathyroid development, in 18% of the kindreds [10]. In our FIHP cohort we identified *MEN1* and *CDC73* mutations in 20% and 7%, respectively. Further molecular studies on FIHP kindreds are warranted in order to discover novel genes involved in their pathogenesis.

The strengths of this study involve: i) collection of the largest series of consecutive patients with hereditary PHPT having full clinical, biochemical, instrumental and genetic characterization, mostly followed at a single Italian institution; ii) inclusion of a large cohort of sporadic MEN1 cases; iii) comparison between the clinical characteristics of patients with sporadic and familial MEN1 syndrome; iv) genotype-phenotype correlation between *MEN1*-positive and *MEN1*-negative patients. However, our study does have some limitations: i) difficulty in assessing the true incidence of *de novo* mutations in the sporadic MEN1 cohort, due to unavailability of genetic data of proband’s parents; ii) possible referral bias for the unusually high frequency of non-functioning pituitary tumors in our series due to our Endocrine Unit also being a referral center for pituitary disease; iii) lack of *GCM2* gene mutational analysis in FIHP kindreds.

In conclusion, the co-occurrence of the three major MEN1-related manifestations, namely PHPT, GEP and pituitary tumors, or only parathyroid and GEP tumors, with or without the presence of minor MEN1-related manifestations, represents the best predictor of a *MEN1*positive test. Although the low rate of *MEN1* mutations in patients with S-MEN1 might raise the suspicion of a phenocopy, we suggest performing *MEN1* genetic analysis, even in the lack of family history of MEN1, in order to diagnose a heritable condition in about half of the index case’s offspring. Further studies searching for alternative genes responsible for MEN1 phenocopies and FHIP are strongly advisable.

## Supporting information

S1 Table Systematic review of published sporadic MEN1 cases with MEN1 gene mutational analysis results and associated phenotype. (DOCX)
