# Supplementary material for: Mutational and large deletion study of genes implicated in hereditary forms of primary hyperparathyroidism and correlation with clinical features
Source: PLoS One. 2017 Oct 16;12(10):e0186485. doi: 10.1371/journal.pone.0186485 (PMC5643132; doi:10.1371/journal.pone.0186485)
Supplement: S1 Table — (DOCX) [file pone.0186485.s001.docx]

**S1 Table. Systematic review of published sporadic MEN1 cases with MEN1 gene mutational analysis results and associated phenotype**

| **S-MEN1 cases (n= 466)** | | | ***MEN1*-positive phenotype (n= 156)** | | | | | | ***MEN1*-negative phenotype (n=310)** | | | | | | **Authors** |
| --- | --- | --- | --- | --- | --- | --- | --- | --- | --- | --- | --- | --- | --- | --- | --- |
| **Total cases (Atypical cases)** | ***MEN1* mutation-positive (Atypical mutation-positive)** | **% *MEN1* mutation-positive (% Atypical *MEN1* mutation-positive )** | **Triad (n=61)** | **PHPT + GEP NET**  **(n=52)** | **PHPT + Pit (n=27)** | **GEP NET + Pit (n=3)** | **Atypical (n=12)** | **na**  **(n=2)** | **Triad (n=27)** | **PHPT + GEP NET (n=49)** | **PHPT + Pit (n=165)** | **GEP NET + Pit (n=14)** | **Atypical**  **(n=55)** | **na**  **(n=0)** |  |
| 11 (3) | 8 (1) | 73 (33) | 2 (PHPT/ZE/GH-PRL, PHPT/I/PRL) | 4 (ZE/I) | 1 (PRL) |  | 1 (PHPT/L /Le/T) |  |  |  | 1 (GH) |  | 2 (PHPT /L, PHPT /Ad) |  | Agarwal et al. [1] |
| 1 (0) | 1 | 100 |  |  | 1 (na) |  |  |  |  |  |  |  |  |  | Mayr et al. [2] |
| 14 (3) | 10 (1) | 82 (33) |  | 1 (na) | 8 (na) |  | 1 (PHPT/Ca) |  | 2 (na) |  |  |  | 2 (PIT/Ad) |  | Giraud et al. [3] |
| 4 (0) | 4 | 100 | 1 (PHPT/Gl/NF) | 3 (G, I, na) |  |  |  |  |  |  |  |  |  |  | Tanaka et al. [4] |
| 2 (0) | 2 | 100 |  | 2 (na) |  |  |  |  |  |  |  |  |  |  | Sato et al. [5] |
| 1 (0) | 1 | 100 | 1 (PHPT/I/NF) |  |  |  |  |  |  |  |  |  |  |  | Hamaguchi et al. [6] |
| 15 (0) | 6 | 40 | 5 (na) |  | 1 (na) |  |  |  | 3 (na) | 1 (na) | 5 (na) |  |  |  | Poncin et al. [7] |
| 7 (0) | 0 | 0 |  |  |  |  |  |  | 1 (na) | 3 (na) | 2 (na) | 1 (na) |  |  | Dackiw et al. [8] |
| 1 (0) | 1 | 100 | 1 (PHPT/I/PRL) |  |  |  |  |  |  |  |  |  |  |  | Cebrian et al. [9] |
| 7 (0) | 1 | 12,5 | 1 (PHPT/I, G/PRL) |  |  |  |  |  |  | 1 (I) | 5 (1 ACTH, 1 NF, 1 PRL, 2 GH) |  |  |  | Bergman et al. [10] |
| 15 (7) | 10 (6) | 67 (86) |  |  | 2 (PRL, na) |  | 6 (2 PNET/Ad, 4 PHPT/Ca) | 2 |  | 1 (na) | 3 (PRL) |  | 1 (PHPT/Ad) |  | Roijers et al. [11] |
| 20 (0) | 8 | 40 | 3 (PHPT/ I/NF, PHPT/NF/ NF, PHPT/G/PRL) | 4 (na) | 1 (PRL) |  |  |  | 1 (I/PRL) | 2 (I, hG) | 8 (6 GH, 1 NF, 1 PRL) | 1 (G/GH) |  |  | Hai et al. [12] |
| 4 (0) | 3 | 75 | 2 (I/PRL, NF/PRL) |  | 1 (GH) |  |  |  |  |  | 1 (na) |  |  |  | Jakobovitz-Picard et al. [13] |
| 1 (0) | 1 | 100 |  | 1 (Gl) |  |  |  |  |  |  |  |  |  |  | Sugiura et al. [14] |
| 1 (0) | 1 | 100 |  | 1 (I) |  |  |  |  |  |  |  |  |  |  | Hai et al. [15] |
| 2 (0) | 2 | 100 | 2 (PHPT/NF/NF, PHPT/I/PRL) |  |  |  |  |  |  |  |  |  |  |  | Park et al. [16] |
| 6 (0) | 3 | 50 | 2 (PHPT/G/GH-PRL, PHPT/na/GH-PRL) | 1 (I) |  |  |  |  |  |  | 3 (1 GH, 2 PRL) |  |  |  | Tso et al. [17] |
| 1 (0) | 1 | 1 |  | 1 (NF) |  |  |  |  |  |  |  |  |  |  | Balogh et al. [18] |
| 1 (0) | 0 | 0 |  |  |  |  |  |  |  |  | 1 (PRL) |  |  |  | Nishimura et al. [19] |
| 1 (1) | 0 (0) | 0 (0) |  |  |  |  |  |  |  |  |  | 1 (PHPT/Ad) |  |  | Honda et al. [20] |
| 80 (10) | 22 (0) | 27,5 (0) | 9 (na) | 10 (na) | 3 (na) |  |  |  | 4 (na) | 7 (na) | 34 (na) | 3 (na) | 10 (6 PHPT/na, 4 PIT/na) |  | Ellard et al. [21] |
| 20 (0) | 6 | 30 | 1 (na) | 5 (na) |  |  |  |  |  | 4 (na) | 10 (na) |  |  |  | Klein et al. [22] |
| 1 (1) | 1 (1) | 100 (100) |  |  |  |  | 1 (NF PIT/Ca) |  |  |  |  |  |  |  | Snabboon et al. [23] |
| 71 (16) | 18 (1) | 25,3 (6,25) | 7 (nd) | 5 (na) | 3 (na) | 2 (na) | 1 (PHPT/Me) |  | 6 (na) | 7 (na) | 21 (na) | 4 (na) | 15 (5 PHPT/Ad, 1 PHPT/Ca, 1 PNET/Ad, 7 Pit/Ad, 1 Pit/Me) |  | Odou et al. [24] |
| 59 (0) | 5 | 8,5 | 4 (PHPT/G/PRL, PHPT/PP/PRL, 2 PHPT/I/PRL) | 1 (PP) |  |  |  |  | 9 (na) | 18 (na) | 27 (na) |  |  |  | Tham et al. [25] |

| **S-MEN1 cases** | | | ***MEN1*-positive phenotype** | | | | | | ***MEN1*-negative phenotype** | | | | | | **Authors** |
| --- | --- | --- | --- | --- | --- | --- | --- | --- | --- | --- | --- | --- | --- | --- | --- |
| **Total cases (Atypical cases)** | ***MEN1* mutation-positive (Atypical mutation-positive)** | **% *MEN1* mutation-positive (% Atypical *MEN1* mutation-positive )** | **Triad** | **PHPT + GEP NET** | **PHPT + Pit** | **GEP NET + Pit** | **Atypical** | **na** | **Triad** | **PHPT + GEP NET** | **PHPT + Pit** | **GEP NET + Pit** | **Atypical** | **na** |  |
| 22 (9) | 3 (0) | 14 (0) | 1 (PHPT/I/GH-PRL) | 1 (na) |  | 1 (I/PRL) |  |  | 1 (na) | 1 (na) | 6 (3 NF, 2 ACTH, 1 GH) | 2 (I/NF, G/GH) | 9 (4 PHPT/Ad, 1 PHPT/Pg, 1 PNET/Ad, 3 Pit/Ad) |  | Balogh et al. [26] |
| 16 (0) | 0 | 0 |  |  |  |  |  |  |  |  | 16 (6 GH, 6 PRL, 2 NF) |  |  |  | Ozawa et al. [27] |
| 2 (0) | 1 | 50 |  | 1 (G) |  |  |  |  |  |  |  | 1 (I/ACTH) |  |  | Peppa et al. [28] |
| 16 (8) | 0 (0) | 0 (0) |  |  |  |  |  |  |  |  | 7 (3 GH, 2 PPRL, 1 NF) | 1 (na) | 8 (1 PHPT/Ca, 3 PHPT/Ad, 4 Pit/Ca) |  | Igreja et al. [29] |
| 1 (1) | 0 (0) | 0 (0) |  |  |  |  |  |  |  |  |  |  | 1 (PNET/Ad) |  | Vierimaa et al. [30] |
| 8 (0) | 4 | 50 |  | 1 (na) | 3 (na) |  |  |  |  | 2 (na) | 2 (na) |  |  |  | Kihara et al. [31] |
| 1 (1) | 1 (1) | 100 (100) |  |  |  |  | 1 (PHPT/Ca) |  |  |  |  |  |  |  | Otake et al. [32] |
| 1 (0) | 1 | 100 |  |  | 1 (ACTH) |  |  |  |  |  |  |  |  |  | Boguszweski et al. [33] |
| 1 (0) | 1 | 100 | 1 (PHPT/NF/PRL) |  |  |  |  |  |  |  |  |  |  |  | Griniatsos et al. [34] |
| 1 (0) | 1 | 100 |  | 1 (na) |  |  |  |  |  |  |  |  |  |  | Kikuchi et al. [35] |
| 14 (5) | 0 (0) | 0 (0) |  |  |  |  |  |  |  | 1 (na) | 8 (na) |  | 5 (3 I/Ad, 2 Ca/Ad) |  | Malanga et al. [36] |
| 10 (2) | 3 (0) | 30 | 1 (PHPT/na/PRL) | 2 (I, Gl, na) |  |  |  |  |  | 1 (I) | 4 (2 GH, PRL, GH-PRL) |  | 2 (PHPT/Ca) |  | Giacchè et al. [37] |
| 1 (0) | 0 (0) | 0 (0) |  |  |  |  |  |  |  |  | 1 (Gl) |  |  |  | Erdas et al. [38] |
| 13 (1) | 13 (1) | 100 (100) | 8 (3 PHPT/NF/PRL, 3 PHPT/I/PRL, 1 PHPT/NF/NF, 1 PHPT/G/NF) | 4 (3 NF, 1 V) |  |  | 1 (PHPT, Hp) |  |  |  |  |  |  |  | Chung et al. [39] |
| 1 (0) | 1 | 100 |  |  | 1 (na) |  |  |  |  |  |  |  |  |  | Jeong et al. [40] |
| 1 (0) | 1 | 100 |  | 1 (G) |  |  |  |  |  |  |  |  |  |  | Birla et al. [41] |
| 8 (0) | 8 | 100 | 6 (2 PHPT/I/PRL, 1 PHPT/I/ACTH, 1 PHPT/G/NF, 1 PHPT/G/PRL, 1 PHPT/NF/ACTH ) | 1 (I) | 1 (PRL) |  |  |  |  |  |  |  |  |  | Goroshi et al. [42] |
| 1 (0) | 1 | 100 | 1 (PHPT/VIP/PRL) |  |  |  |  |  |  |  |  |  |  |  | Cavalli et al. [43] |
| 1 (0) | 1 | 100 |  | 1 (NF) |  |  |  |  |  |  |  |  |  |  | Ohara et al [44] |
| 1 (0) | 1 | 100 | 1 (PHPT/I/NF) |  |  |  |  |  |  |  |  |  |  |  | Itoh & Saikawa [45] |

S-MEN1= Sporadic MEN1 syndrome, PHPT= Primary hyperparathyroism, GEP NET= Gastro-entero-pancreatic neuroendocrine tumors, Pit= Pituitary tumors, ZE= Zollinger-Ellison Syndrome, G= Gastrinoma, Gl= Glucagonoma, I= Insulinoma, NF= Non-functional tumor, VIP= VIPoma, Pg= Paraganglioma, GH= Growth hormone-secreting tumor, PRL= Prolactin-secreting tumor, ACTH= Adrenocorticotropic hormone-secreting tumor, Ad= Adrenal tumor, L= Lipoma, Le= Leiomioma, T= Thyroid tumor, Me= Mesothelioma, Ca= Carcinoid, Hp= Hyperplasia, hG= Hypergastrinemia, na= Not available

**References**

1. Agarwal SK, Kester MB, Debelenko L V, Heppner C, Emmert-Buck MR, Skarulis MC, et al. Germline mutations of the MEN1 gene in familial multiple endocrine neoplasia type 1 and related states. Hum Mol Genet. 1997;6: 1169–75. doi:10.1093/hmg/6.7.1169

2. Mayr B, Apenberg S, Rothamel T, von zur Muhlen A, Brabant G. Menin mutations in patients with multiple endocrine neoplasia type 1. Eur J Endocrinol. 1997;137: 684–687.

3. Giraud S, Zhang CX, Serova-Sinilnikova O, Wautot V, Salandre J, Buisson N, et al. Germ-line mutation analysis in patients with multiple endocrine neoplasia type 1 and related disorders. Am J Hum Genet. 1998;63: 455–67. doi:10.1086/301953

4. Tanaka C, Yoshimoto K, Yamada S, Nishioka H, II S, Moritani M, et al. Absence of germ-line mutations of the multiple endocrine neoplasia type 1 (MENⅠ) gene in familial pituitary adenoma in contrast to MENⅠ in Japanese. J Clin Endocrinol Metab. 1998;83: 960–965. doi:10.1210/jcem.83.3.4653

5. Sato M, Matsubara S, Miyauchi A, Ohye H, Imachi H, Murao K, et al. Identification of five novel germline mutations of the MEN1 gene in Japanese multiple endocrine neoplasia type 1 (MEN1) families. J Med Genet. 1998;35: 915–9. doi:10.1136/jmg.35.11.915

6. Hamaguchi K, Nguyen D, Yanase T, Ikuyama S, Goto K, Takayanagi R, et al. Novel germline mutations of the MEN1 gene in Japanese patients with multiple endocrine neoplasia type 1. J Hum Genet. 1999;44: 43–47. doi:10.1007/s100380050105

7. Poncin J, Abs R, Velkeniers B, Bonduelle M, Abramowicz M, Legros JJ, et al. Mutation analysis of the MEN1 gene in Belgian patients with multiple endocrine neoplasia type 1 and related diseases. Hum Mutat. 1999;13: 54–60. doi:10.1002/(SICI)1098-1004(1999)13:1<54::AID-HUMU6>3.0.CO;2-K

8. Dackiw APB, Cote GJ, Fleming JB, Schultz PN, Stanford P, Vassilopoulou-Sellin R, et al. Screening for MEN1 mutations in patients with atypical endocrine neoplasia. Surgery. 1999;126: 1097–1104. doi:10.1067/msy.2099.101376

9. Cebrián A, Herrera-Pombo JL, Díez JJ, Sánchez-Vilar O, Lara JI, Vázquez C, et al. Genetic and clinical analysis in 10 Spanish patients with multiple endocrine neoplasia type 1. Eur J Hum Genet. 1999;7: 585–589. doi:10.1038/sj.ejhg.5200336

10. Bergman L, Teh B, Cardinal J, Palmer J, Walters M, Shepherd J, et al. Identification of MEN1 gene mutations in families with MEN 1 and related disorders. Br J Cancer. 2000;83: 1009–1014. doi:10.1054/bjoc.2000.1380 S0007092000913806 [pii]

11. Roijers JFM, De Wit MJ, Van Der Luijt RB, Ploos Van Amstel HK, Höppener JWM, Lips CJM. Criteria for mutation analysis in MEN 1-suspected patients: MEN 1 case-finding. Eur J Clin Invest. 2000;30: 487–492. doi:10.1046/j.1365-2362.2000.00664.x

12. Hai N, Aokis N, Shlmatsu A. Clinical features of multiple endocrine neoplasia type 1 (MEN1) phenocopy without germline MEN1 gene mutations: analysis of 20 Japanese sporadic cases with MEN1. Clin Endocrinol. 2000;52: 509–518.

13. Jakobovitz-Picard O, Olchovsky D, Berezin M, Ghodsizade A, Zahavi Z, Karasik A, et al. Mutation analysis of the MEN1 gene in Israeli patients with MEN1 and familial isolated hyperprolactinemia. Hum Mutat. 2000;16: 269. doi:10.1002/1098-1004(200009)16:3<269::AID-HUMU11>3.0.CO;2-2

14. Sugiura H, Morikawa T, Itoh K, Ono K, Okushiba S, Kondo S, et al. Thymic carcinoid in a patient with multiple endocrine neoplasia type 1: report of a case. Surg Today. 2001;31: 428–432.

15. Hai N, Muto G, Okamoto H, Tamada A, Abe R, Suzuki S, et al. A novel germline mutation of the MEN1 gene, L259del, in a patient with sporadic multiple endocrine neoplasia type 1 (MEN1). Jpn J Clin Oncol. 2001;31: 125–7.

16. Park J-H, Kim I-J, Kang HC, Lee S-H, Shin Y, Kim K-H, et al. Germline mutations of the MEN1 gene in Korean families with multiple endocrine neoplasia type 1 (MEN1) or MEN1-related disorders. Clin Genet. 2003;64: 48–53. doi:10.1034/j.1399-0004.2003.00091.x

17. Tso AW, Rong R, Lo CY, Tan KC, Tiu SC, Wat NM, et al. Multiple endocrine neoplasia type 1 (MEN1): genetic and clinical analysis in the Southern Chinese. Clin Endocrinol. 2003;59: 129–135. doi:1812 [pii]

18. Balogh K, Patocs A, Majnik J, Varga F, Illyes G, Hunyady L, et al. Unusual presentation of multiple endocrine neoplasia type 1 in a young woman with a novel mutation of the MEN1 gene. J Hum Genet. 2004;49: 380–386. doi:10.1007/s10038-004-0163-2

19. Nishimura Y, Yamashita K, Yumita W, Yamazaki M, Katai M, Sakurai A, et al. Multiple endocrine neoplasia type 1 with unusual concomitance of various neoplastic disorders. Endocr J. 2004;51: 75–81. doi:10.1507/endocrj.51.75

20. Honda M, Tsukada T, Horiuchi T, Tanaka R, Yamaguchi K, Obara T, et al. Primary hyperparathyroidism associatiated with aldosterone-producing adrenocortical adenoma and breast cancer: relation to MEN1 gene. Intern Med. 2004;43: 310–314. doi:10.2169/internalmedicine.43.310

21. Ellard S, Hattersley AT, Brewer CM, Vaidya B. Detection of an MEN1 gene mutation depends on clinical features and supports current referral criteria for diagnostic molecular genetic testing. Clin Endocrinol (Oxf). 2005;62: 169–175. doi:10.1111/j.1365-2265.2005.02190.x

22. Klein RD, Salih S, Bessoni J, Bale AE. Clinical testing for multiple endocrine neoplasia type 1 in a DNA diagnostic laboratory. Genet Med. 2005;7: 131–138. doi:10.1097/01.GIM.0000153663.62300.F8

23. Snabboon T, Plengpanich W, Siriwong S, Wisedopas N, Suwanwalaikorn S, Khovidhunkit W, et al. A novel germline mutation, 1793delG, of the MEN1 gene underlying multiple endocrine neoplasia type 1. Jpn J Clin Oncol. 2005;35: 280–282. doi:10.1093/jjco/hyi080

24. Odou M-F, Cardot-Bauters C, Vantyghem M-C, Carnaille B, Leteurtre E, Pigny P, et al. Contribution of genetic analysis in screening for MEN1 among patients with sporadic disease and one or more typical manifestation. Ann Endocrinol (Paris). 2006;67: 581–7. doi:AE-12-2006-67-6-0003-4266-101019-200607477

25. Tham E, Grandell U, Lindgren E, Toss G, Skogseid B, Nordenskjöld M. Clinical testing for mutations in the MEN1 gene in Sweden: A report on 200 unrelated cases. J Clin Endocrinol Metab. 2007;92: 3389–3395. doi:10.1210/jc.2007-0476

26. Balogh K, Hunyady L, Patocs A, Gergics P, Valkusz Z, Toth M, et al. MEN1 gene mutations in Hungarian patients with multiple endocrine neoplasia type 1. Clin Endocrinol (Oxf). 2007;67: 727–734. doi:10.1111/j.1365-2265.2007.02953.x

27. Ozawa A, Agarwal SK, Mateo CM, Burns AL, Rice TS, Kennedy PA, et al. The parathyroid/pituitary variant of multiple endocrine neoplasia type 1 usually has causes other than p27Kip1 mutations. J Clin Endocrinol Metab. 2007;92: 1948–1951. doi:10.1210/jc.2006-2563

28. Peppa M, Boutati E, Kamakari S, Pikounis V, Peros G, Koutsodontis G, et al. Novel germline mutations of the MEN1 gene in Greek families with multiple endocrine neoplasia type 1. Clin Endocrinol (Oxf). 2009;70: 75–81. doi:10.1111/j.1365-2265.2008.03308.x

29. Igreja S, Chahal HS, Akker SA, Gueorguiev M, Popovic V, Damjanovic S, et al. Assessment of p27 (cyclin-dependent kinase inhibitor 1B) and aryl hydrocarbon receptor-interacting protein (AIP) genes in multiple endocrine neoplasia (MEN1) syndrome patients without any detectable MEN1 gene mutations. Clin Endocrinol (Oxf). 2009;70: 259–264. doi:10.1111/j.1365-2265.2008.03379.x

30. Vierimaa O, Villablanca A, Alimov A, Georgitsi M, Raitila A, Vahteristo P, et al. Mutation analysis of MEN1, HRPT2, CASR, CDKN1B, and AIP genes in primary hyperparathyroidism patients with features of genetic predisposition. J Endocrinol Invest. 2009;32: 512–518. doi:10.3275/6107

31. Kihara M, Miyauchi A, Ito Y, Yoshida H, Miya A, Kobayashi K, et al. MEN1 gene analysis in patients with primary hyperparathyroidism: 10-year experience of a single institution for thyroid and parathyroid care in Japan. Endocr J. 2009;56: 649–656. doi:10.1507/endocrj.K08E-265

32. Otake Y, Aoki M, Nakanishi T, Hashimoto K. Atypical carcinoid of thymus associated with multiple endocrine neoplasia syndrome type 1. Gen Thorac Cardiovasc Surg. 2010;58: 534–537. doi:10.1007/s11748-009-0557-3

33. Boguszewski CL, Bianchet LC, Raskin S, Nomura LM, Borba LA, Cavalcanti TC. Application of genetic testing to define the surgical approach in a sporadic case of multiple endocrine neoplasia type 1. Arq Bras Endocrinol Metab. 2010;54: 705–710.

34. Griniatsos JE, Dimitriou N, Zilos A, Sakellariou S, Evangelou K, Kamakari S, et al. Bilateral adrenocortical carcinoma in a patient with multiple endocrine neoplasia type 1 (MEN1) and a novel mutation in the MEN1 gene. World J Surg Oncol. 2011;9: 6. doi:10.1186/1477-7819-9-6

35. Kikuchi R, Mino N, Okamoto T, Matsukura T, Hirai T. Simultaneous double thymic carcinoids: A rare initial manifestation of multiple endocrine neoplasia type 1. Gen Thorac Cardiovasc Surg. 2011;59: 68–72. doi:10.1007/s11748-010-0606-y

36. Malanga D, De Gisi S, Riccardi M, Scrima M, De Marco C, Robledo M, et al. Functional characterization of a rare germline mutation in the gene encoding the cyclin-dependent kinase inhibitor p27Kip1 (CDKN1B) in a Spanish patient with multiple endocrine neoplasia-like phenotype. Eur J Endocrinol. 2012;166: 551–560. doi:10.1530/EJE-11-0929

37. Giacché M, Panarotto A, Mori L, Daffini L, Tacchetti MC, Pirola I, et al. A novel menin gene deletional mutation in a little series of Italian patients affected by apparently sporadic multiple endocrine neoplasia type 1 syndrome. J Endocrinol Invest. 2012;35: 124–128. doi:10.1007/BF03345419

38. Erdas E, Aste N, Pilloni L, Nicolosi A, Licheri S, Cappai A, et al. Functioning glucagonoma associated with primary hyperparathyroidism: multiple endocrine neoplasia type 1 or incidental association? BMC Cancer. BMC Cancer; 2012;12: 614. doi:10.1186/1471-2407-12-614

39. Chung YJ, Hwang S, Jeong JJ, Song SY, Kim SH, Rhee Y. Genetic and epigenetic analysis in korean patients with multiple endocrine neoplasia type 1. Endocrinol Metab. 2014;29: 270–9. doi:10.3803/EnM.2014.29.3.270

40. Jeong YJ, Oh HK, Bong JG. Multiple endocrine neoplasia type 1 associated with breast cancer: A case report and review of the literature. Oncol Lett. 2014;8: 230–234. doi:10.3892/ol.2014.2144

41. Birla S, P Jyotsna V, Singla R, Tripathi M, Sharma A. Impact of a novel 14 bp MEN1 deletion in a patient with hyperparathyroidism and gastrinoma. Endocrinol Diabetes Metab Case Reports. 2015;2015: 150011. doi:10.1530/EDM-15-0011

42. Goroshi M, Bandgar T, Lila AR, Jadhav SS, Khare S, Shrikhande S V., et al. Multiple endocrine neoplasia type 1 syndrome: single centre experience from western India. Fam Cancer. 2016;15: 617–624. doi:10.1007/s10689-016-9891-7

43. Cavalli T, Giudici F, Santi R, Nesi G, Brandi ML, Tonelli F. Ventricular fibrillation resulting from electrolyte imbalance reveals vipoma in MEN1 syndrome. Fam Cancer. 2016;15: 645–649. doi:10.1007/s10689-016-9906-4

44. Ohara N, Kaneko M, Ikeda M, Ishizaki F, Suzuki K, Maruyama R, et al. Lung adenocarcinoma and adrenocortical carcinoma in a patient with multiple endocrine neoplasia type 1. Respir Med Case Reports. 2017;20: 77–81. doi:10.1016/j.rmcr.2016.12.002

45. Itoh M, Saikawa Y. A novel MEN1 mutation in a Japanese adolescent with multiple endocrine neoplasia type 1. 2017;26: 25–28.
